# Measuring total liver function on sulfur colloid SPECT/CT for improved risk stratification and outcome prediction of hepatocellular carcinoma patients

**DOI:** 10.1186/s13550-016-0212-9

**Published:** 2016-06-27

**Authors:** Stephen R. Bowen, Tobias R. Chapman, Joshua Borgman, Robert S. Miyaoka, Paul E. Kinahan, Iris W. Liou, George A. Sandison, Hubert J. Vesselle, Matthew J. Nyflot, Smith Apisarnthanarax

**Affiliations:** Department of Radiation Oncology, University of Washington School of Medicine, 1959 NE Pacific St, Box 356043, Seattle, WA 98195 USA; Department of Radiology, University of Washington School of Medicine, 1959 NE Pacific St, Box 356043, Seattle, WA 98195 USA; Department of Physics, University of Washington, 3910 15th Ave. NE, Seattle, WA 98195 USA; Division of Gastroenterology, Department of General Internal Medicine, University of Washington School of Medicine, 1959 NE Pacific St, Seattle, WA 98195 USA

**Keywords:** SPECT/CT, Sulfur colloid, Child-Pugh, ROC, HCC, Liver function

## Abstract

**Background:**

Assessment of liver function is critical in hepatocellular carcinoma (HCC) patient management. We evaluated parameters of [^99m^Tc] sulfur colloid (SC) SPECT/CT liver uptake for association with clinical measures of liver function and outcome in HCC patients.

**Methods:**

Thirty patients with HCC and variable Child-Turcotte-Pugh scores (CTP A5-C10) underwent [^99m^Tc]SC SPECT/CT scans for radiotherapy planning. Gross tumor volume (GTV), anatomic liver volume (ALV), and spleen were contoured on CT. SC SPECT image parameters include threshold-based functional liver volumes (FLV) relative to ALV, mean liver-to-spleen uptake ratio (L/S_mean_), and total liver function (TLF) ratio derived from the product of FLV and L/S_mean_. Optimal SC uptake thresholds were determined by ROC analysis for maximizing CTP classification accuracy. Image metrics were tested for rank correlation to composite scores and clinical liver function parameters. Image parameters of liver function were tested for association to overall survival with Cox proportional hazard regression.

**Results:**

Optimized thresholds on SC SPECT were 58 % of maximum uptake for FLV, 38 % for L/S_mean_, and 58 % for TLF. TLF produced the highest CTP classification accuracy (AUC = 0.93) at threshold of 0.35 (sensitivity = 0.88, specificity = 0.86). Higher TLF was associated with lower CTP score: TLF_A_ = 0.6 (0.4–0.8) versus TLF_B_ = 0.2 (0.1–0.3), *p* < 10^−4^. TLF was rank correlated to albumin and bilirubin (|*R*| > 0.63). Only TLF >0.30 was independently associated with overall survival when adjusting for CTP class (HR = 0.12, 95 % CI = 0.02–0.58, *p* = 0.008).

**Conclusions:**

SC SPECT/CT liver uptake correlated with differential liver function. TLF was associated with improved overall survival and may aid in personalized oncologic management of HCC patients.

## Background

The accurate assessment of liver function is of critical importance in the management of patients with hepatocellular carcinoma (HCC) [1]. The majority of patients with HCC have associated chronic liver disease (viral hepatitis, alcohol injury, or steatosis) [2] and resultant cirrhosis that places patients at risk of morbidity and mortality from liver-directed therapies [3–7]. Traditionally, severity of chronic liver disease has been graded using the Child-Turcotte-Pugh (CTP) classification system [8, 9], originally designed over 50 years ago to estimate mortality in patients with cirrhosis and portal hypertension prior to undergoing surgical treatment of portal hypertension. The CTP system [9] assigns points to 3 continuous objective variables (serum bilirubin, albumin, and prothrombin time) and 2 subjective variables (ascites and hepatic encephalopathy). These points are added together to generate a composite score to assign patients into one of the three classes with increasing liver dysfunction: A, B, or C.

While the CTP classification system has been shown to be an independent prognostic factor, it has inherent limitations that can under- or overestimate the degree of liver dysfunction, such as empiric cutoff values, equal weighting of variables, and challenges with the subjective interpretation of ascites and encephalopathy [10]. More recently, other prognostic models of cirrhosis have been designed, including the model for end-stage liver disease (MELD) score [11, 12] and its derivatives [10], that are utilized for prioritization of liver transplantation. These systems, however, were not specifically designed for patients with HCC, unlike the recently published albumin-bilirubin (ALBI) grade [13]. Staging systems that incorporate both tumor and liver function parameters for HCC patients include the Barcelona Clinic Liver Cancer (BCLC), Cancer of the Liver Italian Program (CLIP), and Japan Integrated Scoring system (JIS) [14]. However, all of these classification systems and models (including CTP and MELD) still rely on traditional measures of liver function, only provide a *global* assessment of liver function, and/or do not take into account spatial variations in liver function [15]. Knowledge of this patient-specific liver function heterogeneity may be valuable in the local therapeutic management of HCC patients [16, 17].

Quantitative imaging of liver function holds promise as an alternative to traditional measures of liver function, if properly validated. Diagnostic imaging of liver function includes positron emission tomography (PET) with [^18^F]fluorodeoxygalactose [18, 19] and dynamic contrast-enhanced magnetic resonance imaging [20] with gadoxetic acid [21] or gadoxetate disodium [22, 23]. Single photon emission computed tomography (SPECT) of several radiotracers have been investigated as surrogates for liver function [18, 19, 21, 24], most notably [^99m^Tc] hepatobiliary iminodiacetic acid [25], [^99m^Tc] galactosyl-human serum albumin [24], and [^99m^Tc] sulfur colloid (SC). The latter is a well-established FDA-approved diagnostic tracer that images the reticuloendothelial (Kupffer) cells of the liver and has been shown to correlate with chronic liver disease severity and function [26–29]. However, the reliability of quantitative molecular imaging parameters as surrogates of global and regional liver function and their relationship to conventional liver function parameters remains unclear. Historical methods of qualitatively or semi-quantitatively assessing SC distribution with 2-D planar scintigraphy provided limited contrast and spatial resolution that relied on expert observer interpretation. Subjective visual scoring of planar scintigraphy was ineffective in detecting variations in liver function [30], which highlights the critical need for more sensitive tests with standardized quantitative parameters.

**Fig. 1:**
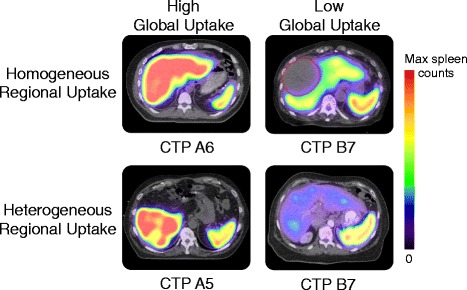
Sulfur colloid SPECT/CT uptake patterns in hepatocellular carcinoma patients with different Child-Turcotte-Pugh score. Four patients are shown with varying degrees of global liver uptake relative to spleen and regional uptake variation, which may be a consequence of their clinical liver function status. Imaging can parameterize these uptake patterns and provide non-invasive longitudinal biomarkers of liver function. Color scales have been normalized to spleen uptake to allow inter-patient comparison of liver uptake. Gross tumor is outlined in *red* where indicated

The evolution of combined SPECT/CT technology has improved regional contrast between functional and non-functional liver from tomographic image acquisition. It has also increased the accuracy in local SC uptake estimation from collimator-detector response modeling, scatter correction, and CT-based attenuation correction, resulting in distinct uptake patterns between patients with differential liver function (Fig. 1). In this era of modern SC SPECT/CT imaging, however, it is not known which imaging metrics are most closely associated with clinical liver function and patient outcomes. The purpose of this study, therefore, is to test measures of [^99m^Tc]SC SPECT/CT liver uptake for statistical association with traditional clinical measures of liver function and survival in HCC patients. Automatic SPECT region-of-interest definition was first optimized to differentiate between CTP classes, and then, SC SPECT/CT metrics were independently correlated to clinical outcome as part of univariate and multivariate analyses. In the absence of absolute quantitative SPECT characterization, relative image intensity ratios were constructed to quantify both magnitude and volumetric extent of liver function.

## Methods

### Patient characteristics

After obtaining Institutional Review Board approval, 30 HCC patients (19 males, 11 females) with a median age of 67 years (range 43–84) were included in the study, from 2013 to 2015. Patients were selected for external beam radiotherapy after multi-disciplinary assessment for suitability and ineligibility for other liver-directed therapies. Twenty-eight patients had unifocal HCC lesions, and gross tumor volumes (GTV) ranged from 1–1573 cm^3^ (median 15 cm^3^). The majority of patients (71 %) had received prior liver-directed therapy (LDT) for HCC and presented with persistent disease, local recurrence, or new HCC lesions, as defined by either contrast enhanced CT or MRI. Prior LDTs included radiofrequency ablation (*n* = 7), transarterial chemoembolization (*n* = 15), radioembolization (*n* = 3), or bland embolization (*n* = 2), and exactly half of all patients received more than one LDT. The median time from the last LDT was 3 months (range 1–35 months). All patients had underlying clinically diagnosed cirrhosis, predominantly with either well-compensated or mildly decompensated liver function: CTP A (*n* = 16), CTP B (*n* = 12), and CTP C (*n* = 2), respectively (range A5–C10). Cirrhosis was related to either hepatitis C (*n* = 17), alcohol intake (*n* = 8), non-alcoholic fatty liver disease (*n* = 5), hepatitis B (*n* = 6), or a combination of these factors (*n* = 7). A minority of patients presented with hepatic encephalopathy (*n* = 6) or ascites (*n* = 5).

CTP class was calculated using history and physical examination findings, imaging results, and laboratory data [8, 9]. Patients presented with a range of baseline albumin (2.1–4.0 g/dL), bilirubin (0.3–2.4 mg/dL), and international normalized ratios (INR) (0.9–5.8). Portal hypertension was defined as clinical or radiographic evidence of portal venous system varices/splenomegaly or ongoing medical management for varices. Splenomegaly was defined when the greatest oblique superior to inferior dimension on sagittal CT scan was >15 cm.

### SPECT/CT image acquisition, reconstruction, and registration

All patients with a history of cirrhosis and/or prior liver-directed therapy treated in our department from 2013 to 2015 underwent [^99m^Tc]SC SPECT/CT scans prior to definitive radiotherapy as part of their clinical care and were reproducibly immobilized in treatment position. SPECT/CT images were acquired on a Precedence™ (Philips Healthcare, Andover, MA) scanner comprising a dual head gamma camera and 16 slice CT scanner. Following the injection of 7 mCi (259 MBq) [^99m^Tc]SC, SPECT scans were acquired 15 min post-injection over a fixed time-averaged frame (64 views, 20 s/view, 180° arc). Emission images were corrected for scatter, collimator-detector response, and attenuation using a tidal breathing end-exhale position CT image. Reconstructions were performed with the Astonish™ (Philips Healthcare, Andover, MA) ordered subset expectation-maximization (OSEM) iterative algorithm over 2 iterations and 16 subsets that included a 10-mm Hanning filter and 4.7 mm isotropic voxels.

Liver anatomy from the end-exhale attenuation correction CT acquired with each SPECT scan was registered to the reference liver anatomy from the end-exhale respiratory phase of a radiotherapy planning CT acquired under free-breathing (*n* = 13), abdominal compression (*n* = 10), or active breathing control (ABC™, Elekta Inc., Stockholm, Sweden) breath-hold conditions (*n* = 7). Rigid registration between the planning CT and SPECT/CT was performed in MIM 6.4™ (MIM Software Inc., Cleveland, OH) using built-in mutual information methods. The resulting spatial transformations estimated from CT-to-CT registration were applied to the respective SPECT images. Deformable registration techniques between the radiotherapy planning CT and attenuation correction CT were initially evaluated but did not provide sufficiently improved liver registration accuracy, particularly in the context of end-exhale CT scans and low spatial resolution SPECT, to warrant their implementation for this study.

### SC SPECT metrics

Regions of interest such as the GTV, anatomic liver volume (ALV, defined as the liver minus GTV), and spleen were defined on the planning CT, with the assistance of contrast-enhanced CT scans as needed. On the spatially aligned SC SPECT image, two relative metrics of functional liver volume (FLV) and liver function magnitude were calculated: the ratio of an uptake threshold-defined functional volume to ALV and the ratio of mean liver counts within the ALV to mean spleen counts (L/S_mean_). These parameters did not rely on absolute quantitative estimates of tracer activity concentration (MBq/mL). Ratios of uptake in the liver versus spleen have been found useful in prior reports [28] and assume that the majority of SC uptake is in the liver and spleen, even in the presence of shunting to the bone marrow in cirrhotic patients. The FLV ratio represents a *volumetric measure* of liver function, while L/S_mean_ represents a *global magnitude* of liver function. A macroparameter calculated from product of the FLV and L/S_mean_ ratios, termed the total liver function (TLF) ratio, is a measure of *integral* liver function. These SC SPECT parameters were calculated by the following equations:

1 $$ \mathrm{F}\mathrm{L}\mathrm{V} = \mathrm{f}\mathrm{V}/\mathrm{A}\mathrm{L}\mathrm{V} $$

2 $$ \mathrm{L}/{\mathrm{S}}_{\mathrm{mean}}={I}_{\mathrm{mean}}^{\mathrm{liver}}/{I}_{\mathrm{mean}}^{\mathrm{spleen}} $$

3 $$ \mathrm{T}\mathrm{L}\mathrm{F} = \mathrm{F}\mathrm{L}\mathrm{V}\kern0.5em \times \kern0.5em \mathrm{L}/{\mathrm{S}}_{\mathrm{mean}} $$

The functional volume (fV) was defined by an image intensity threshold as a percentage of maximum SC SPECT image intensity within the ALV. The mean image intensity (*I*_mean_) was also defined by a fixed threshold as a percentage of maximum SC SPECT image intensity within the ALV or the spleen. A range of image intensity thresholds were tested, as described in the “Statistical analysis” section. The ensemble of SC SPECT uptake measures (FLV, L/S_mean_, TLF) is analogous to the quantitative FDG PET parameters of metabolic tumor volume (MTV), mean tumor uptake (SUV_mean_) or mean tumor-to-blood ratio (T/B_mean_), and total lesion glycolysis (TLG).

### Statistical analysis

Optimal image thresholds for SC SPECT parameter association with CTP classification (A vs. B/C class) were interrogated by receiver-operator characteristic (ROC) analysis. A range of image intensity thresholds to define functional liver regions of interest, calculated as a percentage of maximum SC SPECT liver uptake from 20 to 70 % in increments of 2 %, were included in ROC analysis of FLV ratio, L/S_mean_ ratio, and TLF ratio to maximize the ROC area under the curve (AUC). The maximum AUC image thresholds defined functional liver regions of interest and the SC SPECT metrics for all remaining statistical analysis. Significant differences in Wilcoxon rank sum of ROC-optimized SC SPECT parameters were tested between CTP classes. Spearman rank correlations between SC SPECT parameters and clinical liver function parameters were tabulated for the following categories: (1) composite liver function scoring systems (CTP score, ALBI grade), (2) individual quantitative liver function components (albumin, bilirubin, INR), and (3) individual qualitative clinical liver function components (encephalopathy, portal hypertension, ascites, and splenomegaly). Qualitative components were treated as binary categorical variables (yes/no).

Imaging and clinical parameters of liver function were tested for association with overall survival. Continuous variables were first dichotomized above and below a threshold value that maximized accuracy of classification under ROC with balanced sensitivity and specificity. Univariate Cox proportional hazard regression was then performed on all continuous and categorical variables. Statistically significant variables that were not highly correlated were selected for multivariate Cox proportional hazard regression to maximize model log-likelihood. For this preliminary investigation in a limited patient cohort, one imaging variable and one clinical variable were selected. The simple multivariate model served to adjust for the effect size of established clinical liver function scoring systems (CTP, ALBI) with known association with overall survival. All statistical calculations were performed in OriginPro 9.1™ (OriginLab Corporation, Northampton, MA), and all hypothesis testing reported two-tailed *p* values.

## Results

**Fig. 2:**
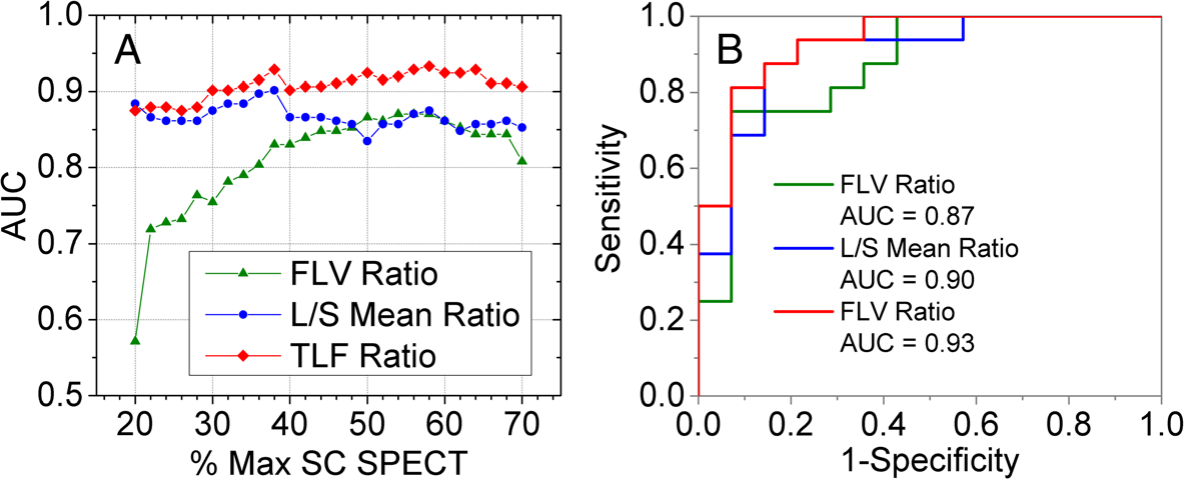
Optimal sulfur colloid SPECT/CT uptake threshold region-of-interest definition for liver imaging parameter prediction of clinical liver function defined by Childs-Turcotte-Pugh A versus B/C class. **a** The receiver operator characteristic (ROC) area under the curve (AUC) is plotted versus the percentage maximum sulfur colloid SPECT uptake threshold used to define three imaging metrics: functional liver volume (FLV) ratio of total liver volume, mean liver-to-spleen (L/S) uptake ratio, and their product as the total liver function (TLF) ratio. **b** ROC curves at the optimal SC SPECT threshold for FLV ratio, L/S mean ratio, and TLF ratios. The product of liver function magnitude and volume yields the highest accuracy for CTP classification, with a TLF cutoff value of 0.35 having a sensitivity of 0.88 and specificity of 0.86

Figure 2 displays the ROC analysis for determination of optimal SC SPECT image uptake thresholds and image parameter for CTP class differentiation (A vs. B/C). As image thresholds of percentage maximum SC SPECT uptake were increased (Fig. 2a), the FLV ratio AUC increased monotonically before reaching a global maximum at 58 %. The L/S_mean_ ratio remained relatively constant in its classification accuracy of CTP outside of a global maximum at 38 %. The TLF ratio as a product of FLV and L/S_mean_ ratios maximized classification accuracy at a threshold of 58 %. The optimal thresholds for each parameter (58 % max SC SPECT uptake for FLV and TLF ratios, 38 % max SC SPECT uptake for L/S_mean_) defined a set of ROC curves in Fig. 2b, which show equal increase in AUC from 0.87 to 0.93. Among these three image parameters, the TLF ratio at the optimal 58 % threshold was best at predicting CTP class with an AUC of 0.93. For an operating point that equalizes specificity and sensitivity, the optimal cutoff values were an FLV ratio of 0.40 (sensitivity = 0.75, specificity = 0.71), L/S_mean_ ratio of 0.88 (sensitivity = 0.88, specificity = 0.86), and TLF ratio of 0.35 (sensitivity = 0.88, specificity = 0.86).

Table 1 lists the difference in the optimized image threshold parameters between CTP classes. The FLV ratio displayed the lowest sensitivity in median difference between CTP A and B/C patients, whereas the L/S_mean_ ratio showed the highest sensitivity in median difference but also the highest intra-class quartile range. The TLF ratio as their product demonstrated both high sensitivity in median difference but low variability in intra-class quartile range, reflected in the lowest probability of a type I error.

**Table 1:** Liver function imaging parameter comparison between Child-Turcotte-Pugh A versus B/C patients

	CTP A (*n* = 16)	CTP B/C (*n* = 14)	Classification cutoff	Rank sum *p*
Functional liver volume ratio (of liver-GTV)	0.5 (0.4–0.6)	0.3 (0.2–0.4)	0.40	6 × 10^−4^
Mean liver-to-spleen uptake ratio	1.4 (0.9–1.6)	0.7 (0.6–0.8)	0.88	2 × 10^−4^
Total liver function (FLV * L/S mean)	0.6 (0.4–0.8)	0.2 (0.1–0.3)	0.35	6 × 10^−5^

Median (interquartile range) values are reported for functional liver volume (FLV), mean liver-to-spleen uptake ratio, and total liver function for Child-Turcotte-Pugh class A and B/C groups. Statistically significant differences between CTP classes were estimated by Wilcoxon rank-sum testing (*α* = 0.05)

**Fig. 3:**
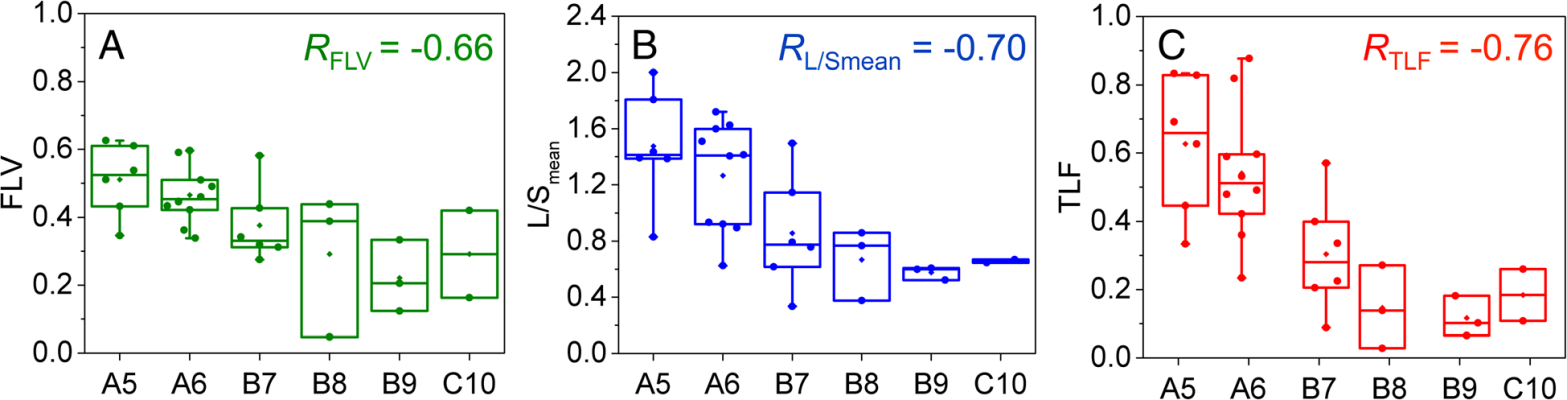
*Box-whisker* plots of sulfur colloid SPECT/CT parameter rank correlation to Child-Turcotte-Pugh score. Functional liver volume (FLV) ratio of total liver volume correlation (**a**), liver-to-spleen mean uptake ratio (L/S_mean_) correlation (**b**), and total liver function (TLF) product (**c**) correlation to Child-Turcotte-Pugh score. TLF product shows the best statistical correlation to CTP score

From the optimized image thresholds, the FLV ratio, L/S_mean_ ratio, and TLF ratio were rank correlated to clinical liver function parameters in Fig. 3. With decreasing clinical liver function (increasing CTP score), all three ratios also decreased. FLV ratio had low variability for patients with CTP A5-B7 but was highly variable for patients with CTP B8-C10. L/S_mean_ ratio had higher variability but decreased monotonically with increasing score from CTP A5-B9. TLF ratio had the most significantly negative rank correlation with CTP score (*R*_TLF_ = −0.76) compared to the other imaging parameters (*R*_FLV_ = −0.66, *R*_L/Smean_ = −0.70). Note that while the inter-quartile ranges in TLF ratio at each CTP score were narrow, the overall range of TLF ratios was especially high at the interface between CTP A6 and B7. This discordance between SC SPECT uptake measures and CTP score for certain patients may be addressed with improved liver function classification models.

**Fig. 4:**
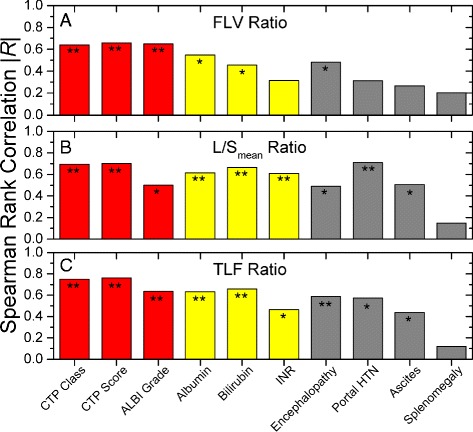
Spearman rank correlations (absolute value) between SC SPECT parameters and clinical parameters of liver function. Functional liver volume (FLV) ratio of total liver volume correlation (**a**), liver-to-spleen mean uptake ratio (L/S_mean_) correlation (**b**), and total liver function (TLF) product (**c**) correlation to composite liver function classification systems (*red*), quantitative liver function parameters (*yellow*), and qualitative liver function parameters (*gray*). *p < 0.01, **p < 0.001

Figure 4 illustrates absolute Spearman rank correlation coefficients as a function of several clinical liver function parameters, grouped by composite scoring systems (red), individual quantitative parameters (yellow), and individual qualitative parameters (gray). FLV ratio was highly correlated to both CTP and ALBI composite scoring systems, but only somewhat correlated to albumin, bilirubin, and encephalopathy. L/S_mean_ ratio was strongly correlated to individual quantitative components (albumin, bilirubin, INR) and portal hypertension but was less correlated to the ALBI grade. TLF ratio had the highest absolute rank correlation to the composite scoring systems (|*R*_TLF_| = 0.64–0.76) and all components (|*R*_TLF_| > 0.4) with the exception of splenomegaly.

The survival analysis was conducted on the patient cohort at a median follow-up post-treatment of 17 months (range 1–29 months), with 12 deaths considered as events and survivors right-censored at the last follow-up. Among survivors, the minimum follow-up interval was 9 months and median follow-up interval was 22 months. Patients who died presented with baseline median TLF = 0.22 (interquartile range = 0.09–0.48). ROC analysis for classification of overall survival yielded a cutoff value of TLF = 0.30 (sensitivity = 0.88, specificity = 0.75). Table 2 lists the variables with statistically significant association to overall survival (OS), including hazard ratios (HR, 95 % confidence intervals) and Cox proportional hazard *p* values. TLF as a dichotomous variable (TLF > 0.30) was most strongly associated to OS (HR = 0.08, *p* < 0.001). For comparison, TLF was dichotomized by a simple median cutoff (TLF > 0.38), under which statistical significance of univariate Cox regression was maintained (HR = 0.20, *p* = 0.02).

**Table 2:** Predictors of overall survival under univariate Cox proportional hazard regression

	HR	95 % CI	Cox *p*
FLV (continuous)	0.005	0–0.64	0.02
L/S_mean_ (>0.81)	0.19	0.05–0.42	0.008
TLF (continuous)	0.03	0.001–0.60	0.02
TLF (>0.30)	0.08	0.02–0.34	<0.001
CTP class	6.21	1.63–23.59	0.007
CTP score	1.73	1.18–2.55	0.005
ALBI grade	4.24	1.28–14.04	0.02

Hazard ratios (HR) and 95 % confidence intervals (CI) are reported along with Cox test of significance. Continuous variables were dichotomized by a threshold value that maximized accuracy under ROC classification with balanced sensitivity/specificity

Multivariate Cox regression was performed by selecting a single imaging parameter (TLF > 0.30) with the highest level of significance and greatest hazard ratio deviation from unity. This prevented inclusion of imaging variables with high cross-correlation. The single imaging variable was paired with a single clinical variable with significant and substantial association to overall survival, either CTP class or ALBI grade, which produced two equivalent multivariate models, listed in Table 3, with log-likelihood *p* < 0.001. After adjusting for clinical scoring systems of liver function (CTP class, ALBI grade), only TLF ratio above/below a threshold of 0.30 was independently associated with overall survival (model A: HR = 0.12, 95 % CI = 0.02–0.58, *p* = 0.008; model B: HR = 0.10, 95 % CI = 0.02–0.42, *p* = 0.002).

**Table 3:** Independent predictors of overall survival under multivariate Cox proportional hazard regression

Model A	HR	95 % CI	Cox *p*
TLF (>0.30)	0.12	0.02–0.58	0.008
CTP class	2.80	0.58–13.56	0.20
Model B	HR	95 % CI	Cox *p*
TLF (>0.30)	0.10	0.02–0.42	0.002
ALBI grade	2.04	0.52–8.00	0.30

Statistically significant univariate predictors (one imaging variable, one clinical variable) were paired. Hazard ratios (HR) and 95 % confidence intervals (CI) are reported along with Cox test of significance. Two equivalent multivariate regression models were generated (model A: comparison to CTP class; model B: comparison to ALBI grade; log likelihood *p* < 0.001)

## Discussion

Parameters of sulfur colloid SPECT/CT have potential to measure liver function and further refine risk stratification models for HCC patients. This investigation sought to establish SC SPECT/CT image parameters that best correlate to clinical liver function by optimizing the automatic thresholding of SC SPECT and correlating relative ratios to composite and individual clinical parameters of liver function. The total liver function (TLF) as a product of liver function magnitude (L/S_mean_) and volume (FLV) was found to be the best imaging classifier of clinical liver function as a result of having the highest accuracy and lowest probability of a type I error in differentiating CTP class. TLF was most correlated with quantitative variables of liver function (albumin, bilirubin), including independent correlation to the recent ALBI composite scoring system. Correlation to qualitative clinical parameters such as portal hypertension was driven by the liver-to-spleen ratio rather than functional liver volume.

Survival analysis revealed that TLF was associated with overall survival after adjusting for CTP class or ALBI grade. Following independent validation of this preliminary finding, TLF may be considered as a candidate imaging biomarker that complements traditional liver function classification systems, particularly in cases of discordant findings. These results support further investigation on the incorporation of complementary volumetric functional imaging into HCC risk stratification models, which would enable quantification of global liver function and characterization of spatial heterogeneity in liver function. The latter is indicated in settings where prior liver-directed therapies may have compromised regions of liver function. Such liver function imaging parameters may become essential for targeted liver therapy planning of external beam radiotherapy, radioembolization, chemoembolization, and surgical resection.

TLF is analogous in construction to total lesion glycolysis from cancer imaging with FDG PET/CT, which in several disease sites has been shown to better predict clinical outcome and survival than imaging parameters of uptake magnitude (SUV_mean_) and metabolic tumor volume (MTV) alone [31–37]. Past investigations have characterized liver function with SC image parameters of magnitude (liver-to-spleen ratio) [28, 29] or size/density (perfused hepatic mass) [26, 27], but the impact of liver dysfunction on oncology management of HCC patients and post-therapy toxicity may be best parameterized by integrating function with the liver volume. When assessing our patient cohort with L/S_mean_ and FLV alone, eight patients (27 %) had image parameters that resulted in different CTP classification (i.e., CTP A using L/S_mean_ and CTP B using FLV and vice versa), which reinforces a complex interplay between volume and magnitude of liver function. The TLF represents a functional liver mass that takes into account both the density and volumetric extent of liver function in patients. This concept of a functional image-defined hepatic mass is intuitively in agreement with surgical literature on the future liver remnant whose functional changes are closely tied to post-surgical outcomes [38–40]. TLF, when strategically paired with quantitative clinical parameters, may prove valuable for prediction of hepatic toxicity and long-term outcome following validation in a larger patient cohort.

Several limitations of this investigation should be noted: (1) sample size: the limited number of events and patients in this preliminary survival analysis prevented definitive multivariate Cox regression with cross-validation to power testing on independent patient cohorts but rather supported the identification of TLF as a candidate imaging biomarker; (2) variability in contouring of the normal liver on CT: despite a single radiation oncologist (S.A.) defining the anatomic liver volume region of interest, variation introduced by manual contouring methods can affect the denominator of the functional liver volume ratio; (3) standardization of imaging protocols: while a fixed threshold can be optimized for a particular imaging protocol at a single institution, any lack of protocol standardization that changes reconstruction algorithms or filters introduces a bias in the estimate of image parameters and propagates these errors across multiple institutions. Infrastructure and resources for establishing and standardizing quantitative FDG PET/CT parameters [41, 42] should also be adapted for quantitative imaging of liver function; (4) relative quantitation of SPECT/CT: uncertainty in modeling of photon collimator blurring, coupled with uncertainty in scatter and attenuation correction, have historically limited the absolute quantitative accuracy of SPECT imaging [43]. Advances in SPECT image reconstruction that incorporate improved physics modeling suggest an achievable quantitative accuracy with errors of less than 5 % [44]. This study bridges the gap between qualitative assessment and absolute quantitation of tracer uptake by SPECT imaging through definition of semi-quantitative relative metrics. Future investigations will explicitly calculate tracer uptake that has been standardized to provide absolute quantitative accuracy and precision characterization, which may improve quantitative imaging-based prediction of liver function; (5) the anatomic liver volume was defined on a radiotherapy planning CT acquired during a separate patient exam whereas functional liver parameters were defined on the sulfur colloid SPECT. Co-registration of these images can further introduce unwanted variation in liver morphology between the scans and bias the volume estimates. These sources of error in the FLV ratio may explain the lower degree of correlation to several clinical liver function parameters, although in general, the imaging parameters were defined as relative ratios to mitigate inter-patient variability in region-of-interest definition; and lastly, (6) SC SPECT/CT images Kupffer cells but not hepatocytes and may provide an indirect measure of liver function. Despite this possibility, prior large prospective studies have shown that imaging of Kupffer cells as measured by perfused hepatic mass independently predicts long-term clinical outcomes in patients with chronic liver disease [26] and is correlated with indocyanine green (ICG) clearance in plasma [28], a validated quantitative measure of global liver function [45].

Our data revealed several discordances between clinical parameters and imaging parameters of liver function. Of the individual clinical liver function parameters tested, splenomegaly had the poorest correlation to imaging parameters. The dependence of splenomegaly assessment on spleen size could in principle affect the liver-to-spleen ratio, but no statistical association was observed. One possible explanation for a lack of correlation is the non-standardized method for identifying clinical splenomegaly, which relies on either subjective evaluation or an arbitrary size cutoff, and is therefore prone to variability in visual perception. Such qualitative parameters have been removed from recent scoring systems like ALBI, which further reinforces the value in quantitative functional imaging.

Moreover, there was a subset of patients who had discordances between liver function as defined by TLF and clinically defined portal hypertension, a patient characteristic that is often used by surgeons as a relative contraindication for therapeutic surgical resection due to concerns of post-resection-related liver failure and morbidity. Of the patients with documented portal hypertension, 8 of 18 patients had TLF ratios above the cutoff value of 0.30 associated with improved overall survival, suggesting that up to 44 % of patients could be considered for surgical resection depending on the extent of planned resection [46]. This implication is hypothesis-generating and would need to be validated in a larger cohort of patients. However, the dissociation of portal hypertension and SC imaging as seen in our study is not entirely surprising as a prior study demonstrated that the degree of abnormality detected on SC SPECT scans was not necessarily directly due to portal hypertension but related to impaired SC extraction by the reticuloendothelial cells of the liver [47]. To provide additional clarity in the discordance between SC SPECT image parameters and clinical liver function parameters, future work in our group will include integrating other quantitative methods of assessing liver function such as ICG clearance.

The power of SC SPECT/CT imaging resides in the parameterization of liver function spatial heterogeneity. With improvements in SPECT/CT quantitative accuracy [48, 49], come opportunities to perform radiomics analysis [50] of image intensity, image texture [51], and image shape features to characterize functional heterogeneity. Standardization of image acquisition, reconstruction, and segmentation prior to radiomics analysis [52, 53] will be critical to improve reproducibility of radiomic feature estimation [54, 55]. Such high dimensional imaging feature sets will necessitate machine learning algorithms of training/(cross)-validation patient groups for feature selection and model parameter fitting, which may lead to robust risk stratification and outcome prediction of individual patients. It still remains unknown whether liver function radiomics will add clinical utility relative to simple imaging or tissue biomarkers of liver function. Nevertheless, prediction of both global magnitude and regional variation in liver function may critically inform on selection and guidance of liver-directed therapies within the scope of future investigations.

## Conclusions

Sulfur colloid SPECT/CT demonstrated utility through correlation with clinical liver function and association with overall survival in our preliminary investigation of 30 HCC patients undergoing definitive radiotherapy. Following its validation as an imaging biomarker, the total liver function (TLF) ratio may complement existing risk classification schemes of HCC patients to promote precise selection of patients for liver-directed therapies *a priori*, including surgery, targeted intra-arterial embolizations, and even liver transplantation assessment, as well as modify radiotherapeutic strategies to reduce treatment-related liver morbidity and optimize tumor control [17]. Further investigation in a larger patient cohort spanning a wider range of clinical liver function is needed and underway to validate these initial findings.
